# Factors associated with the uptake of biosimilars for breast cancer treatment from the perspectives of physicians and patients-Evidence from China

**DOI:** 10.3389/fphar.2022.1044798

**Published:** 2023-01-12

**Authors:** Qiyou Wu, Zhiwei Lian, Xin Wang, Hanchao Cheng, Jing Sun, Hui Yu, Gong Zhang, Fan Wu, Jian Liu, Chuanben Chen

**Affiliations:** ^1^ School of Health Policy and Management, Chinese Academy of Medical Sciences, Peking Union Medical College, Beijing, China; ^2^ Clinical Oncology School of Fujian Medical University, Fujian Cancer Hospital, Fuzhou, China

**Keywords:** biosimilar, breast cancer, uptake, physician, patient

## Abstract

**Objective:** To investigate the factors associated with the treatment of breast cancer with biosimilars from the perspectives of physicians and patients, and to generate evidence for promoting the uptake of biosimilars.

**Methods:** This study targeted trastuzumab and its indicated human epidermal growth factor receptor 2 (HER2) positive breast cancer and included female HER2 positive breast cancer patients under treatment of trastuzumab at a provincial oncology medical center in southern China from 1 January 2021, to 31 December 2021. The study extracted patients’ demographic, socioeconomic and clinical information and the basic information of their attending physicians from the hospital information system. We performed a bivariate multiple logistic regression analysis of predictive factors of the use of trastuzumab biosimilar.

**Results:** A total of 446 patients (aged ranging between 26 and 74, 51.4 ± 9.06) were included in the analysis, and 19.1% chose biosimilar trastuzumab. Older patients, patients enrolled in the urban and rural resident health insurance program compared with those enrolled in the urban employee health insurance program, patients who initiated treatment after January 2021 when biosimilar entered clinical use compared with those who initiated treatment before, patients with female attending physicians, younger attending physicians and with chief attending physicians compared with deputy chief attending physicians were more likely to adopt biosimilar trastuzumab for treatment (*p* < 05). Controlling the other factors unchanged, when the patient’s attending physician was deputy chief physician, increasing 1 year age of the patient was associated with an increased probability of adopting biosimilar by .8% (*dy/dx* = .008, 95%*CI*: .002–.01, *p* = .01). When the patient was aged between 26 and 60, the probability of adopting biosimilar for the patient whose attending physician was a chief physician was higher than for those whose attending physician was a deputy chief physician, and the gap was the largest when the patient was at the age of 45 (*dy/dx* = .20, 95%CI: .13–.27, *p* < .01).

**Conclusion:** The uptake rate of biosimilars is still low at its initial development stage in China. Educational policies and physicians making recommendations to the indicated patients at the initiation stage of treatment are helpful to avoid reduced willingness to switch to biosimilars due to non-clinical reasons. Patients with lower ability-to-pay will have better accessibility to biologic regimens through the uptake of biosimilars. Official guidelines and professional training are critical to enhancing physicians’ willingness and confidence in adopting biosimilars.

## Introduction

Unlike small-molecular chemical products with well-specified structures, biological agents are macromolecular substances with complex structures and manufacturing processes and are usually highly priced (Antman et al., 2017; Kirchhoff et al., 2017). Taking cancer medicines as an example, biological agents are increasingly highly priced and have a budget impact across countries, casting a shadow over the sustainability of national healthcare systems (Luzzatto et al., 2018; Godman et al., 2021). The need for a better balance between innovation and accessibility arises, hence biosimilars are raised as a potential solution to this dilemma. Biosimilars are similar to reference biologics in terms of quality, safety, and efficacy (Kirchhoff et al., 2017), they are medically equivalent to reference biologics but at a lower cost (Stebbing et al., 2017; Von Minckwitz et al., 2017; Pivot et al., 2018; Triantafyllidi and Triantafyllidis, 2022). Promoting the clinical application of biosimilars could stimulate competition, forcing down prices of reference biologics, thus to help saving health expenses and improving the accessibility of treatment based on biological regimens (Lyman et al., 2018; Chen et al., 2019; Jensen et al., 2020; Mouslim et al., 2020; Lobo and Río-Álvarez, 2021; Moorkens et al., 2021; Okoro, 2021). Higher biosimilar uptake rate is especially significant for low- and middle-income countries, where access to high-cost biological regimens is even worse (Putrik et al., 2014; Baumgart et al., 2019; Gershon et al., 2019; Al-Ziftawi et al., 2021; Tubic et al., 2021).

The European Union (EU) approved the world’s first biosimilar in 2006 and successively implemented a series of policies to promote the uptake of biosimilars, aiming to address the affordability issue of those highly-priced biological agents for both the health systems and individuals (Dylst et al., 2014; Chen et al., 2019; Diao et al., 2019; Jiang et al., 2019; Li et al., 2021a; Li et al., 2021b; Diao et al., 2021; Lobo and Río-Álvarez, 2021; Moorkens et al., 2021; Diao et al., 2022; Godman et al., 2022). These policies include biosimilar substitution (Dong et al., 2019), the requirement of a proportional volume or value of biosimilars prescribed by healthcare providers (Moorkens et al., 2017), financial incentives for clinical use of biosimilars for both patients and healthcare providers associated with health insurance reimbursement and settlement (Costa-Font and Rico, 2006; Berdud et al., 2016; Leonard et al., 2019; Gasteiger and Petrie, 2022). Researches have been conducted to prove the positive effects of these policies on national health systems across Europe, including saved budget for funding new medicines and increasing medicine volumes, gained sustainability of national health insurance system and price reduction of reference biologics (Godman et al., 2020; Godman et al., 2022).

The development of biosimilars in China is later than in the EU, but with a recent surge. China approved the first biosimilar in 2019, and the number of approved biosimilars doubled during the past 3 years with a strong pipeline (Hu et al., 2022). Many studies analyzed the factors associated with the uptake of biosimilars in the countries outside China (Costa-Font and Rico, 2006; Berdud et al., 2016; Alnahar et al., 2017; Mouslim et al., 2020; Lobo and Río-Álvarez, 2021), including physician’s and patient’s knowledge and attitudes towards biosimilars (Tubic et al., 2021), physician’s prescribing habit (Colloca et al., 2019; Okoro, 2021; Krstic et al., 2022), patient’s affordability and experience of treatment with reference biologics (Teeple et al., 2019a). While studies about biosimilars conducted in Chinese settings are mainly limited to clinical research or market entry approval (Diao et al., 2019; Xu et al., 2019; Rahalkar et al., 2021; Ren et al., 2021; Xu et al., 2021), uptake of biosimilars and studies about healthcare providers and patients are rare (Qu et al., 2021; Hu et al., 2022). This study generated evidence based on the real-world clinical data of a provincial oncology center in southern China, expecting to inform decision-making in promoting the uptake of biosimilars from the perspectives of physicians and patients.

## Methods

### Study design

This study was a retrospective analysis based on real-world clinical data. We divided the targeted patients into the reference and biosimilar groups and analyzed the factors associated with biosimilar uptake with a multiple regression analysis.

### Population and data source

The study took trastuzumab as an example for the analysis. The reference trastuzumab was marketed in China in 2002 and covered by the national primary health insurance through national price negotiations since 2017, indicated for adjuvant and neoadjuvant therapy of human epidermal growth factor receptor 2 (HER2) positive breast cancer (China Anti Cancer Association, 2021). The first biosimilar trastuzumab was approved in China in 2020 and was immediately included in the national primary health insurance in 2021. The treatment cost of reference trastuzumab has been continuously reduced through national price negotiations from 2017 to 2019 and is now close to that of the biosimilar trastuzumab. Although there is only one biosimilar trastuzumab marketed in China by the end of July 2022, many applications are under review.

This study included female HER2 positive breast cancer patients under trastuzumab treatment from January 2021 to December 2021 in a provincial oncology center in southern China. The inclusion criteria for the targeted patients were: 1) female; 2) >18 years old; 3) diagnosed as HER2 positive breast cancer; 4) with precise tumor stage (I ∼ IV); 5) under treatment with trastuzumab (either the reference or the biosimilar); 6) under treatment of trastuzumab during January 2021 to December 2021. Patients with trastuzumab treatment contraindications, those with a history of another primary malignancy, and those with incomplete documentation of data were excluded. The inclusion criteria for the attending physicians were: 1) deputy chief or chief physicians; 2) specialized in breast cancer diagnosis and treatment; 3) with continuous clinical practice experiences in the research setting within the past 5 years (2017–2021). Since the target medical center performed team-based management for breast cancer treatment, although other members may give their advice on therapeutic measures, only the deputy chief or chief could be appointed the attending physician and make final decisions including prescription. So we excluded physicians unable to make prescription decisions (with a lower academic rank than the deputy chief) and those who did not prescribe trastuzumab (neither the reference nor the biosimilar) from January 2021 to December 2021. We extracted the targeted patients’ demographic, socioeconomic and clinical information, and their attending physicians’ basic information from the hospital information system.

### Variables

The outcome variable was whether or not biosimilar was used during the observation time (used = 1, not used = 0). The explanatory variables were the characteristics of patients and their attending physicians. Patients’ demographic, socioeconomic and clinical characteristics included age, the economic development level of patient residence, residence in urban or rural areas, type of health insurance coverage, and whether they seek treatment locally. The economic development level of residence was categorized according to the lower and upper quartiles of each area’s annual average *per capita* disposable income (<$5924 as low, $5924–7405 as middle, and >$7405 as high). The clinical characteristics of patients included tumor stage and whether treatment with trastuzumab was initiated before January 2021, when the first biosimilar of trastuzumab started clinical use in the research setting. The characteristics of the attending physicians of patients included gender, age and academic rank.

### Statistical analysis

The study firstly performed a descriptive statistical analysis of the distributions of the included patients’ demographic, socioeconomic and clinical characteristics and the characteristics of their attending physicians. We performed χ^2^ test and Fisher’s exact test to compare the distribution variations of the characteristics between the reference and the biosimilar groups. We had variables with statistically significant distribution variations and those supported by professional judgment included in a binary multiple logistic regression model and analyzed the predictive factors of using biosimilars from the perspectives of both physicians and patients. We also used the ‘margins’ command to analyze the interactive effect of physician and patient on the use of biosimilars. We set the significance level at .05 and completed all statistical analysis with STATA15.1 (Stebbing et al., 2017).

## Results

### Characteristics of patients and attending physicians

The demographic, socioeconomic and clinical characteristics of the included patients are reported in Table 1. A total of 446 patients were included in the analysis; only 85 (19.1%) were treated with the biosimilar. Patients were aged between 26 and 74 (51.4 ± 9.06). Patients diagnosed with early breast cancer (stage I ∼ II) accounted for 63.0% of all cases. Characteristics of attending physicians corresponding to the included patients are reported in Table 2. 24 physicians were included in this study. These physicians were aged between 42 and 62 (51.5 ± 5.89). Among them, 16 were male, and 12 were chief physicians.

**TABLE 1:** Characteristics of the included patients.

Characteristics	Total [n(%)]	Reference group [n(%)]	Biosimilar group [n(%)]	*P* value
	446	361(80.9)	85(19.1)	
Tumor stage				.18
I∼II	281(63.0)	231(64.0)	50(58.8)	
III	76(17.0)	64(17.7)	12(14.1)	
IV	89(20.0)	66(18.3)	23(27.1)	
Age^a^				.02
<47	122(27.4)	108(29.9)	14(16.5)	
47∼56	198(44.4)	159(44.0)	39(45.9)	
>56	126(28.3)	94(26.1)	32(37.6)	
Residence in urban or rural areas				.73
Urban areas	233(52.2)	190(52.6)	43(50.6)	
Rural areas	213(47.8)	171(47.4)	42(49.4)	
The economic development level of the residence				.61
Low	155(34.8)	122(33.8)	33(38.8)	
Middle	142(31.8)	115(31.9)	27(31.8)	
High	149(33.4)	124(34.3)	25(29.4)	
Type of health insurance coverage				.03^b^
Urban employee program	163(36.5)	142(39.3)	21(24.7)	
Urban and rural resident program	278(62.3)	215(59.6)	63(74.1)	
Others (including no insurance)	5(1.2)	4(1.1)	1(1.2)	
Whether seeking medical treatment locally				.71
Yes	181(40.6)	145(40.2)	36(42.4)	
No	265(59.4)	216(59.8)	49(57.6)	
Whether treatment with trastuzumab initiated before January 2021				.13
Yes	53(11.9)	47(13.0)	6(7.1)	
No	393(88.1)	314(87.0)	79(92.9)	
Gender of the attending physician				<.01
Male	344(77.1)	291(80.6)	53(62.4)	
Female	102(22.9)	70(19.4)	32(37.6)	
Age of the attending physician^c^				<.01
<50	135(30.3)	119(33.0)	16(18.8)	
50∼58	196(43.9)	140(38.8)	56(65.9)	
>58	115(25.8)	102(28.3)	13(15.3)	
Academic rank of the attending physician				<.01
Deputy chief physician	125(28.0)	113(31.3)	12(14.1)	
Chief physician	321(72.0)	248(68.7)	73(85.9)	

^a^ Patients’ age groups categorized according to the lower (47) and upper (56) quartiles.

^b^ Fisher’s precision probability test.

^c^ Corresponding physician’s age groups categorized according to the lower (50) and upper (58) quartiles.

**TABLE 2:** Characteristics of the attending physicians.

Characteristics	N (%)
	24
Gender	
male	16(66.7)
female	8(33.3)
Age	
mean (standard deviation)	51.5(5.89)
Academic rank	
deputy chief	12(50.0)
chief	12(50.0)

The distribution variations of patient age (*p* = .02) and type of health insurance coverage (*p* = .03) between the reference and the biosimilar groups were statistically significant; the distribution variations of attending physicians’ gender, age and academic rank (*p* < .01) between the reference and biosimilar groups were statistically significant (Table 1).

### Logistic regression analysis results

Firstly, we included patient variables with statistically significant distribution variations (patient’s age and type of health insurance coverage) and variables supported by professional judgment (tumor stage and whether treatment with trastuzumab initiated before 2021) in the logistic regression model (**Model 1**). The regression results showed that older patients (*p* = .02) and patients enrolled in the urban and rural resident health insurance program compared with those enrolled in the urban employee health insurance program (*p* = .03), were more likely to adopt biosimilar for breast cancer treatment (Table 3).

**TABLE 3:** Logistic regression analysis of the associated factors of biosimilar uptake (*n* = 446).

Variables	Model 1				Model 2			Model 3	
	*OR* (95%*CI*)		*p*-value		*OR* (95%*CI*)		*p*-value	*Β* (95%*CI)*	*p*-value
Patient’s age	1.04(1.01–1.07)		<0.01		1.03(1.01–1.06)		0.02	.12(.04–.20)	<0.01
Tumor stage (Ref. I ∼ II)									
III	.85(.42–1.71)		0.64		.87(.42–1.81)		0.71	−.14(-.87–.59)	0.72
IV	1.73(.95–3.14)		0.07		1.78(.95–3.34)		0.07	.55(-.09–1.18)	0.09
Type of health insurance coverage (Ref. urban employee program)									
urban and rural resident program	1.93(1.11–3.33)		0.02		1.87(1.06–3.29)		0.03	.67(.10–1.24)	0.02
others	1.74(.16–18.7)		0.65		2.18(.17–28.3)		0.56	1.06(-1.53–3.65)	.42
Whether treatment with trastuzumab was initiated before January 2021 (Ref. no)									
yes	.41(.16–1.03)	0.06		.36(.14–.95)		0.04		−1.04(-2.00∼-.09)	0.03
Physician’s age				.91(.86–.97)		<0.01		−.09(-.15∼-.04)	<0.01
Physician’s gender (Ref. male)									
female				2.30(1.31–4.02)		<0.01		.84(.28–1.40)	<0.01
Physician’s academic rank (Ref. deputy chief physician)									
chief physician				4.35(2.01–9.39)		<0.01		1.73(.85–2.61)	<0.01
The interaction term of patients’ age and physicians’ academic rank								−.10(-.19∼-.01)	0.03

Then we included physician variables (physician’s age, gender and academic rank) in the regression model (**Model 2**), and the results showed that patient with younger attending physicians (*p* < .01), female attending physicians compared with the male (*p* < .01), and patients with chief attending physicians compared with the deputy chief attending physicians (*p* < .01) were more likely to adopt biosimilar for breast cancer treatment (Table 3). We also found that patients initiated treatment after January 2021 were more likely to adopt biosimilars compared with those before January 2021 (*p* = .04), which had been on the edge of insignificance in **Model 1** (*p* = .06). Other patient variables in **Model 1** (patient’s age, tumor stage and type of health insurance coverage) remained almost the same in significance and effects in **Model 2**.

In order to explore how the interactions between physician and patient affected the adoption of the biosimilar, we had the characteristic variables of patients and physicians interacted and included in the regression model (**Model 3**). We decentralized the continuous variable (patient’s age). Only the interaction between the patient’s age and the physician’s academic rank was statistically significant (*p* = .03). The regression results of **Model 3** are reported in Table 3. The associated factors in **Model 2** (patient’s age, type of health insurance coverage and whether treatment was initiated before January 2021; physician’s age, gender and academic rank) remained statistically significant in **Model 3** (all *p* < .05).

### Marginal effects of Patient’s age and Physician’s academic rank

**FIGURE 1:**
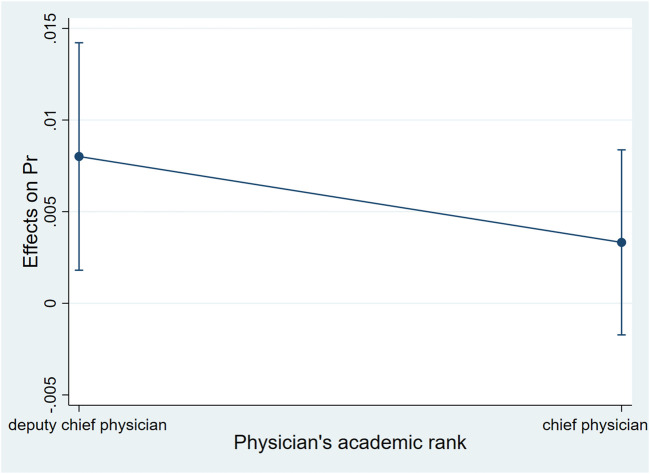
Average marginal effects of patient’s age with 95%*CI*when their attending physicians were with different academic ranks.

We used the ‘margins’ command to precisely estimate the marginal effects of patients’ age interacting with the academic rank of their attending physicians on the probability of adopting biosimilars. Controlling the other factors unchanged, when the academic rank of the attending physician was deputy chief physician, increasing 1 year age of the patient was associated with an increased probability of adopting biosimilar by .8% (*dy/dx* = .008, 95%*CI*: .002–.01, *p* = .01); when the academic rank of the attending physician was chief physician, patient’s age did not have a statistically significant association with the adoption of biosimilar (Table 4; Figure 1).

**TABLE 4:** Fixing the academic rank of the attending physician, the marginal effects of patient’s age on biosimilar uptake.

Academic rank of attending physician	*dy/dx*	95%*CI*	*p*-value
deputy chief physician	.008	.002–.01	.01
chief physician	.003	−.002–.008	.20

**FIGURE 2:**
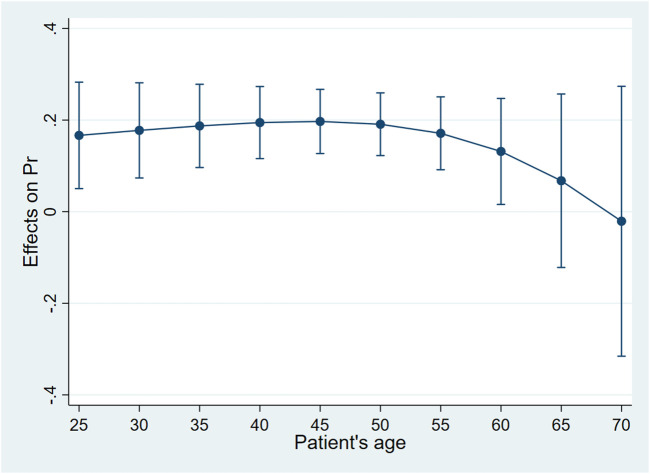
Average marginal effects of attending physician’s academic rank with 95%*CI*when patients were of different ages.

Controlling the other factors unchanged, when the patient was younger than 45 years old, the probability of choosing biosimilar for the patient whose attending physician was a chief physician was higher than for those whose attending physician was a deputy chief physician. Such a gap increased for the elder patient. When the patient was 45 years old, the gap was the largest, and the probability of adopting biosimilar for the patient whose attending physician was a chief physician was 20.0% higher than for those whose attending physician was a deputy chief physician (*dy/dx* = .20, 95%*CI*:0.13–.27, *p* < .01). When the patient was between 45 and 60 years old, the probability of adopting biosimilar for those whose attending physician was a chief physician was still higher than for those whose attending physician was a deputy chief physician. The gap decreased for the elder patient. When the patient reached 60 years old, the academic rank of their attending physician no longer had statistically significant associations with biosimilar uptake (Table 5; Figure 2).

**TABLE 5:** Fixing patient’s age, the marginal effects of chief physician compared with the deputy chief physician on biosimilar uptake.

Patient’s age	*dy/dx*	95%*CI*	*p*-value
25	.17	.05–.28	.01
30	.18	.07–.28	<.01
35	.19	.10–.28	<.01
40	.19	.12–.27	<.01
45	.20	.13–.27	<.01
50	.19	.12–.26	<.01
55	.17	.09–.25	<.01
60	.13	.02–.25	.03
65	.07	−.12–.26	.49
70	−.02	−.32–.27	.89

## Discussion

2021 was the first year that the trastuzumab biosimilar entered clinical use in the research setting. Less than 20% of patients included in this study under trastuzumab treatment adopted biosimilar in this year. At the initial development stage of biosimilars, the uptake rate was much lower in China than in the EU, the latter ranged from 41% to 77% in recent years and has been continuously increasing (Jarrion et al., 2022). Given the rapid development of biosimilars and immediate insurance coverage of the marketed biosimilars, we expect intense competition of trastuzumab and enhanced uptake of biosimilars in China.

This study found that the patient’s age was an essential factor associated with biosimilar uptake. The older the patients were, the higher the probability they would adopt biosimilars. This finding was consistent with the existing evidence (Shelbaya et al., 2021; Yang et al., 2022). Elderly patients may be more price sensitive and tend to use cheaper biosimilars, hoping to avoid a heavier financial burden on themselves or their children. Cancer patients with lower ability-to-pay prioritized lower treatment expenditure over treatment effects, especially for those at the terminal stage. Some patients even rejected treatment to avoid catastrophic expenditure (Meropol et al., 2008; Jenkins et al., 2013; Shrestha et al., 2019). This situation is relatively common in China, where the elderly are usually not the family’s breadwinners, and their out-of-pocket medical expenditures are usually undertaken by their children.

This study also found that only 11.3% of patients who initiated the treatment with reference trastuzumab switched to biosimilar trastuzumab when the later was available. Studies in the UK and the New Zealand also found a lower switching rate for patients who initiated the treatment with the reference product than new patients (Hemmington et al., 2017; Aladul et al., 2018). Physicians may not support switching for non-clinical reasons with a consideration of the potential psychological discomfort of patients (Teeple et al., 2019b). Information asymmetry due to lack of professional training and clinical data disclosure of biosimilars may be another reason for the non-preference of biosimilars. A survey showed that 85% of patients diagnosed with rheumatoid arthritis, Crohn’s disease, ulcerative colitis, psoriasis or psoriatic arthritis did not choose to switch to biosimilars; the primary concern of patients was the safety of biosimilars (Teeple et al., 2019a). Even if the treatment effect may not change significantly or improve after switching, patients may still be resistant to biosimilars when they have already been treated with reference biologics (Jørgensen et al., 2017; Avouac et al., 2022; Jarrion et al., 2022). Such a situation gets worse if adverse effects occur after switching. Patients may mistakenly attribute these symptoms to biosimilars, further exacerbating their mistrust of biosimilars (Gasteiger and Petrie, 2022). The weak intention of switching is a difficult question yet to be solved across countries (Dutta et al., 2020; Allocati et al., 2022). To promote the uptake of biosimilars, one appropriate solution is to enhance educational policies for both patients and physicians. Physicians recommending biosimilars to the indicated patients at the initial stage of treatment may also be available to avoid reduced willingness to switch due to non-clinical reasons. Health authorities could also be of help by formulating clinical guidelines about switching or incentivising biosimilar uptake. Health insurance coverage was associated with the choice of biosimilars. Currently, the Chinese population is universally covered by two parallel primary health insurance programs, the urban employee program and the resident program covering both urban and rural residents. The benefits packages of the former program are stronger than the latter, mainly due to the gaps in the amount of fundraising. The latter is funded heavily relying on the government subsidy for the population with a comparatively lower ability-to-pay. For patients with equivalent ability-to-pay, those enrolled in the urban employee program may be less likely to choose biosimilars than the urban and rural residents. The latter may be more concerned about higher out-of-pocket expenditure constrained by their weaker benefits packages. Physicians usually consider patients’ health insurance coverage, ability-to-pay and other economic factors when setting treatment plans. Thus, physicians are more inclined to recommend cheaper biosimilars to patients covered by a health insurance program with weaker benefits packages and lower ability-to-pay (Sullivan et al., 2017; Cook et al., 2019). Promoting the uptake of biosimilars will help to improve the accessibility of biologic agents for patients, especially for those with weaker health insurance benefits packages and lower ability-to-pay (Diao et al., 2019; Li et al., 2021a; Li et al., 2021b; Diao et al., 2021; Diao et al., 2022).

Our analysis from the perspective of the patient’s attending physician found that older physicians were less likely to have their patients treated with biosimilars. This may be associated with the tendency of older physicians to be more conservative. Conversely, young physicians may be more willing to accept newly developed medical technologies and are more open to follow cutting-edge evidence (Ren et al., 2021; Hu et al., 2022). A study of physicians showed that clinicians over 50 became more conservative about switching to biosimilars (57%); they thought biosimilars were newly marketed, lacking long-term clinical use evidence compared with the references (Leonard et al., 2019). Female physicians were more likely to adopt biosimilars than males. Studies found that female physicians show more empathy as well as a stronger willingness to communicate with patients, so they may be more likely to prescribe biosimilars to reduce medical expenditure or perform better explanations to patients about biosimilar regimens (Cooper-Patrick et al., 1999; Sandhu et al., 2009; Chaitoff et al., 2017). Chief physicians were more likely to adopt biosimilars than deputy chief physicians. This may be because physicians with senior academic ranks may have a better understanding and confidence in biosimilars. Studies showed that physicians with less clinical experience tended to choose more expensive imported medicines to satisfy patients or avoid medical responsibilities caused by poor treatment effects in Chinese healthcare settings (Zhu, 2012; Hu et al., 2022). Foreign studies also reported that physicians with more clinical experience or higher academic ranks were more confident in choosing biosimilars for patients with complex symptoms (Cook et al., 2019; Poon et al., 2021).

Although the price sensitivity of patients and the lower price of biosimilars may be the point of considering switching, this was not always the case like in eastern European countries, where were absent of substitution policy and appropriate incentives for the switch (Tubic et al., 2021). To enhance physicians’ willingness to switch to biosimilars, health authorities and healthcare associations may play an essential role in formulating guidelines for clinical application and switching of biosimilars, as well as strengthening professional training to make physicians fully understand the equivalence and interchangeability of biosimilars. Studies have found that demand-side policies like educational measures and official guidelines could enhance the confidence of physicians in biosimilars by avoiding information asymmetry (Kim et al., 2020; Godman et al., 2022). Financial incentives implemented in the EU to promote the uptake of biosimilars could also be considered in China when more biosimilars are available and more clinical safety and efficacy evidence is generated in the real world.

## Limitations and future perspectives

This study had several limitations. First, this was a single-center study based on observations only 1 year after the availability of the first biosimilar in China. The study only focused on a single indication and one biological agent. The findings should be carefully interpreted for expanded indications and biological products. A small sample size may limit the efficiency of statistical analysis. Secondly, some essential socioeconomic characteristics of both physicians and patients (such as the patient’s educational background, occupation and income, physician’s length of clinical experience and professional training background, *etc.*) may also affect the uptake of biosimilars. With constraints of the retrospective data extracted from the hospital information system in the real clinical world, we were not able to collect information on all these variables. The only available socioeconomic characteristic of patients was insurance coverage. The study calculated each region’s annual average *per capita* disposable income to indicate patients’ ability-to-pay and analyzed the attending physicians’ academic rank to indicate their clinical experience and professional training background. The missing measurement should be considered through comprehensive investigations in future studies.

## Conclusion

The uptake rate of the biosimilar is still low at its initial development stage in China. Educational policies on biosimilars and physicians making recommendations of biosimilars to the indicated patients at the initial stage of treatment are helpful to avoid reduced willingness to switch due to non-clinical reasons. Patients with a weak ability-to-pay will have better accessibility to biological regimens through the uptake of biosimilars. Official guidelines and professional training are critical to enhancing physicians’ willingness and confidence in adopting biosimilars.

## Data Availability

The original contributions presented in the study are included in the article/supplementary materials, further inquiries can be directed to the corresponding author.
